# The Institute for Global Orthopedics and Traumatology: A Model for Academic Collaboration in Orthopedic Surgery

**DOI:** 10.3389/fpubh.2017.00146

**Published:** 2017-06-30

**Authors:** Devin James Conway, Richard Coughlin, Amber Caldwell, David Shearer

**Affiliations:** ^1^University of California San Francisco, San Francisco, CA, United States

**Keywords:** Institute for Global Orthopedics and Traumatology, global health, orthopedic surgery, collaboration, musculoskeletal burden, international orthopedics

## Abstract

In 2006, surgeons at the University of California, San Francisco (UCSF) established the Institute for Global Orthopedics and Traumatology (IGOT), an initiative within the department of orthopedic surgery. The principal aim of IGOT is to create long-term, sustainable solutions to the growing burden of musculoskeletal injury in low- and middle-income countries (LMICs) through academic partnership. IGOT currently has relationships with teaching hospitals in Ghana, Malawi, Tanzania, Nicaragua, and Nepal. The organizational structure of IGOT is built on four pillars: Global Surgical Education (GSE), Global Knowledge Exchange (GKE), Global Research Initiative (GRI), and Global Leadership and Advocacy. GSE focuses on increasing surgical knowledge and technical proficiency through hands-on educational courses. The GKE facilitates the mutual exchange of surgeons and trainees among IGOT and its partners. This includes a global resident elective that allows UCSF residents to complete an international rotation at one of IGOT’s partner sites. The GRI strives to build research capacity and sponsor high-quality clinical research projects that address questions relevant to local partners. The fourth pillar, Global Leadership and Advocacy aims to increase awareness of the global impact of musculoskeletal injury through national and international courses and events, such as the Bay Area Global Health Film Festival. At the core of each tenet is the collaboration among IGOT and its international partners. Over the last decade, IGOT has experienced tremendous growth and maturation in its partnership model based on cumulative experience and the needs of its partners.

## Introduction

Over the last several decades the contributors to global burden of disease have shifted. Injuries as a result of trauma are becoming increasingly common and are now a source of more global DALYs than HIV/AIDs, malaria, and tuberculosis combined. Based on data from the 2013 Global Burden of Disease Study, road injuries alone went from 10th in 2005 to the 7th most common cause of DALYs worldwide (1). There are an estimated 1.3 million deaths from road traffic injuries every year (2), and injuries are the most common cause of death between the ages of 15 and 29 (3). More than 90% of injury-related mortality occurs in low- and middle-income countries (LMICs). While these mortality statistics are staggering, it only begins to address the scope of this problem, as it has been estimated that an additional 20 to 50 million people are disabled by road traffic injuries every year (3). Musculoskeletal injuries, such as femur fractures and open tibia fractures, are substantial but understudied contributor to this burden. In addition, a number of other orthopedic diseases, including osteomyelitis, congenital and acquired limb deformity, and degenerative joint disease are major public health issues that fall within the domain of orthopedic surgeons.

Unfortunately, many developing nations lack the capacity to manage the growing burden of musculoskeletal disease and injury. While models of international collaboration have been effective in other medical specialties, orthopedic surgery has lagged behind its counterparts. Thus, novel frameworks for improving orthopedic capacity in low-resource settings are needed, focusing on long-term sustainability and partner-driven goals. The organizational structure for the Institute for Global Orthopedics and Traumatology (IGOT), an initiative within the Department of Orthopedic Surgery at the University of California, San Francisco (UCSF), is presented here as an academic model that may be replicated at other institutions to improve musculoskeletal care abroad.

## Background and Rationale

While global health has traditionally focused on reducing the burden of infectious disease, there is increasing recognition of the global impact of trauma, including musculoskeletal injuries (4). Primary prevention is commonly touted as the most effective and cost conscious method to reduce disease, and trauma is no exception. Programs aimed to reduce the burden of trauma through increased road traffic safety and regulations have shown some success in LMICs (5, 6). However, even in Europe and the United States, where resources for traffic enforcement, safer roads and vehicles, and other injury prevention programs are immense, emergency medical transport systems and trauma centers play a crucial role in the secondary and tertiary prevention of death and disability from injury. Within trauma centers, orthopedic surgeons have the expertise to maximize function and reduce disability from fractures and dislocations involving the spine, pelvis, and extremities, thereby improving quality of life and helping victims of trauma return to being productive members of society. In addition, a variety of more chronic conditions may benefit from access to musculoskeletal specialists, ranging from pediatric deformities to musculoskeletal infection to degenerative arthritis. Unfortunately, access to high-quality orthopedic care in developing settings is limited by lack of specialist providers and training programs as well as limited infrastructure and material resources, including operating rooms and surgical implants. For example, in 2008, Uganda reported only 23 orthopedic surgeons in the country, 1 for every 1.3 million people (7). At the same time, research to quantify the unmet burden created by these deficiencies and evaluating potential strategies to address them is sorely lacking. A sustainable approach to addressing these barriers is desperately needed.

Orthopedic surgeons have long recognized the need for their expertise in LMICs. There is a rich history of volunteerism in orthopedic surgery, primarily through short-term surgical missions (8). While this model may be effective for a limited number of patients, the bigger picture impact and sustainability are limited once surgeons return to their home institutions. Thankfully this strategy has begun to adapt based on lessons learned from other medical and surgical specialties.

The model of international academic collaboration to address global issues has been well established in many fields of modern medicine. Infectious disease has long been a specialty of medicine associated with overseas collaboration, treating diseases, such as AIDS, malaria, and tuberculosis (9–12). Access to surgery is being increasingly recognized as a global health issue, as the poorest countries in the world—representing a third of the global population—only account for a small percentage of operations performed annually (13, 14). Despite these staggering statistics, academic models of collaboration in surgical fields lag behind their counterparts in medicine. There are a few exceptional models, particularly in pediatric surgery (15–17). However, these programs are almost exclusively within the realm of general surgery. The role of orthopedic surgeons in the broader picture of global health is likely to be unique and has not been well established. While the global need for orthopedics is clear to its practitioners, there is no clear model to address this need, and data supporting one approach relative to another are wholly lacking. Adding to the challenge is that no all-encompassing innovation will be universally adaptable. Rather, each initiative will need to be unique and malleable to fit the needs and locations being addressed (14). Given the need for increased global orthopedic presence without a consensus on effective strategies, IGOT serves an important purpose in both the orthopedic and public health communities. The framework represents a decade of trial and error through frequent communication with international partners to best address the needs they deem most critical. The purpose of this article is to present IGOT and its organizational structure as a potential model for other academic institutions, particularly in orthopedics and other surgical subspecialties, to develop an organized, scalable, and realistic approach to global outreach through collaborative partnerships.

## Case Description

In the summer of 2006, IGOT was founded as an initiative within the department of orthopedic surgery at UCSF (18). The professionals involved in its inception all shared a passion for global health in addition to orthopedics. IGOT’s founders were three orthopedic surgeons with a long track-record of international work, including leadership roles in such organizations as Orthopedic Overseas, the International Committee of the Red Cross, and Medecins sans Frontieres. They shared a vision of an organization dedicated to creating long-term, sustainable solutions to the growing burden of musculoskeletal injury. By focusing on collaboration with institutions around the world—rather than the simple provision of short-term services—the founders hoped to promote a culture of training and investigation to be shared equally among all partners. IGOT was one of the first academic organizations created to address the need for comprehensive orthopedic care on a global scale (18). By fostering academic partnerships in LMICs, IGOT aims to build local capacity for research, education, and clinical care. By focusing on academic centers, impact is maximized through a “train the trainers” model.

## Methodological Aspects

International collaboration is a proven, effective tool for developing health-care capacity in LMICs. However, the framework that comprises these collaborative efforts can be widely variable among different fields of medicine. Moreover, there is little experience with global partnerships in orthopedic surgery. IGOT is one of the first academic organizations to address the global need in orthopedic surgery. Thus, we believe the organizational framework described below represents an adaptable model that can be utilized by other institutions to improve global outreach in orthopedics.

Institute for Global Orthopedics and Traumatology utilizes a four “pillar” structure that categorizes the various modes of successful academic partnership. The four pillars are Global Surgical Education (GSE), Global Knowledge Exchange (GKE), Global Research Initiative (GRI), and Global Leadership and Advocacy. The GSE pillar aims to disseminate knowledge and enhance surgical technique in all fields of orthopedic surgery with an emphasis in orthopedic trauma. This is accomplished through courses—exemplified by the Surgical Management and Reconstructive Training (SMART) course—and online training. The GKE pillar facilitates the bilateral exchange of surgeons and trainees between UCSF and LMICs. This is accomplished through a resident global elective and international fellowships and observerships. The GRI focuses on building capacity to conduct clinical research. By addressing many of the common barriers to conducting research in low-resource settings, the GRI makes high-quality clinical research feasible and thereby facilitates research that addresses locally relevant clinical questions. Finally, the Global Leadership and Advocacy pillar strives to establish the role of orthopedic surgery as a key component of international health. Numerous publications, conferences, and events, such as the Bay Area Global Film Festival, help to increase awareness of this growing problem. Through these four pillars, IGOT aims to have a positive impact on the burden of orthopedic trauma worldwide.

Currently, IGOT has active partnerships with five different hospitals around the world. While some sites function only in the resident exchange program, others feature active and productive collaborative research efforts and recurring educational courses. In Central America, IGOT partners with the El Antonio Lenin Fonseca Hospital in Managua, Nicaragua. In Asia, the only formal partnership is with the Rehabilitation Hospital for Disabled Children in Kathmandu, Nepal. Finally, IGOT has three partnerships in Africa: Komfo Anoyke Teaching Hospital in Kumasi, Ghana, Queen Elizabeth University Teaching Hospital in Blantyre, Malawi, and the Muhimbili Orthopedic Institute (MOI) in Dar es Salaam, Tanzania. IGOT works with surgeons from numerous other sites across the world in the absence of a formal partnership. While the current network is broad, IGOT continues to grow and expand its partnership opportunities. A key tenet in IGOT’s philosophy is the belief that the activities of each collaboration should be driven by the needs of international partners. The programs and courses offered are dynamic and constantly evolving to meet the needs at each partner site. This helps ensure that the curriculum is locally relevant and culturally appropriate to each setting. Unlike short-term surgical missions that focus primarily on clinical care based on the interests of visiting surgeons, IGOTs programs focus on education of local providers through exchange and courses driven by local needs. Research questions are driven by partners and hence generate results with the potential to change practice both locally and globally among surgeons practicing in low-resource environments. The impact, therefore, extends well beyond the individual patient encounters that occur in a typical surgical mission.

### GSE: The IGOT SMART Course

With the growing global burden of injury (1), there is an increasing need for orthopedic services worldwide. Unfortunately, the traumatic injuries encountered are often not limited to bone. Many cases of severe orthopedic trauma also involve extensive soft-tissue injury. Without soft-tissue coverage procedures, such as muscle flaps or skin grafts, this damage can lead to infection, amputation, or even death (19). In high-resource settings, these complex injuries are usually treated using multidisciplinary approach, which typically includes plastic surgeon with specialization in soft-tissue reconstruction to manage the soft-tissue loss. While studies have shown that interventions to address soft-tissue injury in LMICs improve patient outcomes (20), there is a dearth of local surgeons with sufficient training. Similarly, most orthopedic surgeons from HICs on mission trips are similarly unable to address the issue because they are accustomed to having plastic surgeons available for assistance. With this in mind, IGOT has created a cross-disciplinary training approach, hosting an annual course designed to train orthopedic surgeons from low-resource settings how to address complex soft-tissue injuries in addition to providing more conventional training focused on complex fracture management.

A short course teaching soft-tissue reconstruction was first suggested by IGOT’s partners at SIGN Fracture Care International. Soft-tissue management was identified as a critical skill needed for surgeons who, despite having the ability to nail long bones using SIGN implants, were struggling to manage complex open injuries. Initially small “flap courses” were held at Duke and the University of Southern California. In 2010, IGOT hosted its first flap course in San Francisco (SF), which has now become an annual event in conjunction with the SIGN Conference in Richland, Washington. Over time, it was recognized that more surgeons could be reached by conducting the course abroad at partner sites in addition to the annual course in SF. At that point, the modular curriculum encompassing treatment for complex limb injuries became known as the *S*urgical *M*anagement *a*nd *R*econstructive *T*raining (SMART) course.

The SF SMART course is held annually at the Orthopedic Trauma Institute within the Zuckerberg San Francisco General Hospital. This facility provides both lecture and laboratory space to enable didactic as well as hands-on practical education in a cadaver lab. The course focuses on topics, such as wound healing, skin grafts and rotational muscle flaps, and principles of limb reconstruction (21). Surgeons are invited from many different countries to attend the course. Attendees are either funded through their own institution, their Ministry of Health, or occasionally self-funded. In some select cases, IGOT will cover travel costs for attendees. IGOT incurs the cost of hosting the event, such as laboratory costs, food, and transportation to and from accommodations. There is no registration fee to attend.

During the course, participants split time between lectures, small group case-based panel discussions, and skills lab sessions. Lectures are designed to teach the limb-salvaging procedures that will subsequently be performed in the skills lab. These lab sessions take place in a simulated operating room environment and feature fresh cadavers to better replicate an actual surgical setting. Procedures are taught by fellowship-trained plastic surgeons with experience in extremity reconstruction. The curriculum focuses on flaps that can easily performed without loupes, an operating microscope, or microvascular instruments. The major emphasis is on lower leg coverage using the gastrocnemius, soleus, and reverse sural flaps. The SMART course also utilizes case-based panel sessions that engage participants in problem solving complex cases based on the resources available in their settings. Panels are held on a variety of topics, such as the mangled extremity, osteomyelitis, bone loss, as well as other subspecialty topics such as pediatric trauma, bone tumors, and amputations. Attendees have the opportunity to submit relevant cases beforehand to be discussed during the small group panels. This helps to engage participants and ensures the case discussions are relevant to the problems faced by surgeons in LMICs.

Despite the burden of trauma in LMICs, there is a paucity of research that is locally relevant or generalizable from HICs to quantify the burden or establish guidelines for appropriate treatment based on available resources. Few initiatives exist that promote local investigation, despite the growing need for region-specific research. To support the growth of research capacity, IGOT created the International Research Symposium, a 1-day course held in conjunction with the SF SMART course. The course structure is divided into three parts: (1) developing a research question, (2) developing a research protocol, and (3) implementing your research. Each section is comprised of one to two lectures followed by small group discussion. Attendees are encouraged to come to the symposium with a specific research question prepared. The goal is to develop the idea from a non-specific question to a well-developed hypothesis and outline for a study protocol that addresses issues ranging from study design and eligibility to budget and personnel. The International Research Symposium is open to all surgeons attending the SMART course and is typically attended by approximately one-third to one-half of the larger course.

To increase access to the SMART course for surgeons unable to travel to SF, IGOT began hosting an annual “In-Country” course in 2014 at the MOI in Dar es Salaam, Tanzania. The course is modeled after the SF SMART course, but includes additional modules focused on fracture management principles. The most recent course included a separate module focused on deformity management using both acute and gradual correction methods with Ilizarov techniques. The course is attended by approximately 100 surgeons from roughly countries across East and Central Africa. This has greatly expanded the reach of the course for practicing surgeons and trainees who would otherwise be unable to afford travel to SF.

After the success of Tanzania, IGOT has expanded the “In-Country” model to Asia. In November 2016, IGOT conducted an inaugural Nepal SMART course in Kathmandu in collaboration with the National Academy of Medical Sciences of Nepal, the Nepal National Trauma Center (Bir Hospital), and Tribhuvhan University Teaching Hospital. This course was more focused, as all attending surgeons were from Nepal. This partnership developed out of conversations surrounding the Nepal earthquake in April 2015.

In contrast to Tanzania, the course faculty included Nepalese plastic surgeons in addition to international faculty. From the beginning, one of the explicit goals in bringing the SMART course to Nepal has been an emphasis on “training the trainers.” A Memorandum of Understanding was established with the goal of transitioning the SMART course from being externally organized by IGOT to being driven primarily by Nepalese leadership with limited outside support after 3 years. This model builds capacity and allows IGOT to focus resources on fostering new partnerships and expanding the SMART course to other locations.

In 2015, researchers at IGOT published an article detailing some of the successes of the SF SMART course from 2010 to 2014, as well as the 2014 and 2015 Tanzania SMART course (21). The SF SMART course during that time hosted over 200 participants from 25 different countries, while the Tanzania SMART course taught over 100 attendees from 12 different east African countries. A 1-year follow-up was sent to the 2012 SMART course participants detailing flap use. About 77% of surgeons responded. Collectively they reported using 594 flaps, of which they considered 554 to be successful. Overall they reported these flaps to have prevented 121 amputations. Additionally, these surgeons reported disseminating the knowledge to other surgeons and residents, such that an additional 28 surgeons were now performing flap procedures (21). These data demonstrate that the SMART course is an effective method for teaching orthopedic surgeons how to successfully perform flap procedures and prevent limb loss. IGOT intends to use mobile technology to more objectively measure clinical outcomes after flap procedures following the 2016 Nepal SMART course.

To facilitate independent study both pre- and post-course, IGOT has begun creating content for an online education curriculum using the edX platform (22). edX is an open-source software developed by MIT and Harvard that is now used at over 70 universities across the United States to create interactive online courses that include reading material, instructional videos, questions, discussion forums, and robust analytics to evaluate use and knowledge acquisition. The IGOT Portal curriculum will coincide with the curriculum of the SMART course modules, beginning with soft-tissue flaps and limb reconstruction. The portal will also provide a forum to share cases and feedback among users and moderators. Ultimately, this interface will be powerful reinforcement for SMART course attendees.

### GKE: Resident Exchange Program and Visiting Fellowships

International volunteerism has long been an unofficial facet of the orthopedic surgery department at UCSF. Beginning in 1992, residents of the department departed on short-term mission trips to Central and South America through Operation Rainbow. From 1992 until 1998, nearly half of the graduating residents in that timeframe incorporated an international mission into their residency experience. Of these, six continued to volunteer overseas after graduation; of these six, three went on to lead other UCSF residents on international trips (23). In providing residents these global experiences early in their career, the department hoped to instill them with a life-long desire for overseas work.

With the popularity and success of these mission trips, personnel at UCSF sought to expand. It was believed that increasing the duration of the trips and establishing fixed locations would accomplish more than simply provide a meaningful international experience. The increased time would grant residents deeper perspective about the challenges faced by local surgeons working in LMICs. Additionally, having fixed locations would facilitate a more continuous exchange of knowledge among the visiting residents and the local staff members. Thus in 1998, UCSF implemented a 1-month long global health elective for orthopedic surgery residents. This rotation was offered in Umtata, South Africa in conjunction with Orthopedic Overseas. An article published in 2002 stated that 13 out of 17 (76%) of residents had chosen to pursue this elective since its inception (24). The program has continued to remain effective, as residents who take part in the elective are more likely to pursue international work following residency (25).

Though the elective is no longer offered in South Africa, residents now choose among IGOTs five partnerships in Africa, Asia, and Central America. Currently nearing the end of its second decade, the resident global elective remains a unique and vital aspect of the orthopedic residency program at UCSF.

In addition to sending residents overseas, IGOT also provides opportunities for orthopedic surgeons from around the world to visit UCSF. In collaboration with the Orthopedic Trauma Institute at the Zuckerberg San Francisco General Hospital, IGOT offers clinical observerships to provide global leaders in orthopedics a chance to work and learn in SF. These observerships are 2–4 weeks long, and are open to attending physicians, residents, researchers, and medical students. Since 2006, IGOT has hosted over 60 scholars from 20 different countries (26). Often, IGOT will also sponsor visitors to continue their stay in SF following the SMART course. By hosting physicians and researchers from around the world, IGOT hopes to provide valuable experience with surgical practice and research processes at UCSF.

### Global Research Initiative

The GRI seeks to foster and develop research capacity among orthopedic surgeons practicing in LMICs. Despite an overwhelming burden in low-resource settings, the majority of research involving orthopedic trauma is conducted in high-resource environments. In one study, less than 7% of orthopedic research was authored by researchers from developing countries (27). Through collaboration with academic sites around the world, IGOT seeks to foster and support clinical research programs in developing countries. While both resource and labor intensive, these partnerships are very fruitful in producing high-quality research studies. Support ranges from simple feedback on project proposals to intensive collaborative studies supported through study design, funding, short-term personnel, and data analysis. By utilizing teaching hospitals as partner sites, the effect is exponential. Not only are locally relevant questions answered but a culture of research and critical assessment of outcomes is also fostered in an environment that trains future leaders. Our prior work has demonstrated the positive effects of collaboration on the quality of clinical research generated in LMICs (28).

At present, three of the five IGOT partners are actively engaged in major partnership research projects including two randomized controlled trials, one multicenter study, and one long-term prospective cohort study. Partners have twice won the best international presentation at the OTA annual meeting in the last 4 years. Perhaps more importantly, partners have increasingly been presenting and publishing their own works without external assistance, indicating the capacity building nature of the partnership model.

As an example, a major research priority for the GRI has been assessing the value of surgical treatment for femoral shaft fractures, one of the most common and debilitating fractures encountered in LMICs. A large prospective cohort study was recently completed in Tanzania demonstrating that surgical treatment is safe and effective when performed with intramedullary implants rather than plates and screws (29). Secondary studies have also demonstrated the potential cost savings associated with access to efficient surgical treatment and relatively poor outcomes with skeletal traction (30, 31). A multicenter study is currently ongoing in Malawi to establish the cost-effectiveness of surgical treatment compared to skeletal traction. Combined with a current study in Tanzania estimating the country-wide incidence of femur fractures and access to surgery, these data will be able to establish the unmet surgical burden of femoral shaft fractures in LMICs. These data will fill a critical gap and not only influence clinical care, but perhaps more importantly, form the foundation to advocate for increasing access to surgical treatment in LMICs.

Through a generous endowment, IGOT has the capability to support a senior medical student for a 1-year research experience. This opportunity is designed for students interested in orthopedic surgery and global health. Every year, IGOT awards this fellowship to one senior medical student who then becomes involved in a multitude of roles. Foremost, the fellow has an active role in IGOT’s research activities. Responsibilities include the design and implementation of new projects, assisting in the support or analysis of current projects, and the writing of abstracts or manuscripts for completed studies. Additionally, fellows serve as a key component in communications among IGOT and both local and international partners. Finally, the fellow assists with the other various aspects of IGOT operations, from the SMART COURSE and Research Symposium to numerous other events throughout the year. The utilization of senior medical students in this role creates a mutually beneficial relationship. The fellows gain invaluable experience in their anticipated field of interest prior to starting their graduate education, while IGOT acquires an individual who can devote the vast majority of his or her time to research activities. In a field where researchers are usually also practicing surgeons, having a dedicated research personnel grants IGOT a larger capacity to manage projects and pursue additional studies.

### Global Leadership and Advocacy

The final pillar in IGOT’s four-pillar organizational structure is Global Leadership and Advocacy. The purpose of this facet is to raise awareness about the global burden caused by musculoskeletal disease and orthopedic trauma. IGOT accomplishes this through a variety of methods. One example is the annual Bay Area Global Health Film Festival, a film screening event hosted by IGOT that highlights pertinent issues in public health relevant to IGOT’s mission. In addition, members of IGOT actively raise awareness and promote efforts to improve orthopedic care in LMICs through publications, editorials, and conferences year-round. Faculty members also endeavor to find leadership roles in organizations, such as the AAOS International Committee and OTA Humanitarian Committee, among others.

## Discussion

While IGOT now has a decade of experience, many challenges still remain. A significant one is the relative lack of objective, measurable outcomes regarding its interventions. As referenced earlier, available data support the positive impact of the SMART course. However, studies to date are limited to pre- and post-course assessments and self-reported data. Feedback from contributors maintains the overall adaptability of the model and ensures that the interventions remain dynamic. However, long-term outcomes are needed to evaluate the broader impact and sustainability of the model. We are currently utilizing mobile-based data collection tools to record skills taught at the SMART course longitudinally both to measure the effectiveness of the program and provide real-time feedback to course participants.

The financial sustainability is a second major challenge. IGOT functions primarily on grant funding and charitable donations. Grants support has largely been through foundations supporting IGOT’s general mission and educational programs. In addition, the GRI has been supported for specific projects by research funding predominantly from orthopedic organizations, such as the Orthopedic Research and Education Foundation, Orthopedic Trauma Association, AO Foundation, and Foundation for Orthopedic Trauma. A critical next step for IGOT is to successfully compete for grant funding from the NIH and other global health funders that have not historically provided support to orthopedic initiatives.

One final challenge encountered by IGOT is the navigation and growth of international partnerships. While these collaborations are what make this organization successful, the maintenance of these relationships is quite labor intensive, which only increases as new partnerships are created. The most successful programs are typically driven by a champion on each side of the partnership who maintains the relationship, facilitates communication, and ensures that goals are being met. This restricts growth to the number of willing and available champions, which in a busy orthopedic surgery department, often becomes the rate-limiting step for new partnerships and long-term growth.

## Future Recommendations: Expanding the Model

In light of the rapid growth of IGOTs programs over the past decade and demand for new partnerships, efforts are underway to expand the model by replicating it at other institutions. Through a collaborative initiative among personnel at IGOT and orthopedic surgeons across North America, a novel coalition has been created to develop capacity for global orthopedics at multiple academic institutions. The Consortium of Academic Traumatologists (COACT), though still in its organizational infancy, already boasts 20 participating institutions in the United States and Canada. In these partnerships, COACT has developed an extensive network that aims “[t]o support the collaboration of academic global health and orthopedic efforts through mentorship, sharing of best practices, research opportunities, and resources” (32). Through this consortium, investigators at IGOT hope to exchange ideas with like-minded institutions and expand educational and research support more broadly to LMICs in a way that a single university cannot realistically achieve. Based on the lessons and pitfalls from more than a decade of experience, IGOT hopes to share best practices for global partnership in orthopedic surgery that can be replicated. Ultimately, we believe these strategies are a model to address the growing burden of musculoskeletal injury in LMICs in a sustainable manner driven by local needs.

## Author Contributions

DC is the primary author responsible for writing the article. RC and AC were invaluable in contributing information and guidance throughout the manuscript. DS is the senior author responsible for overseeing the manuscript and performing final editing/review.

## Conflict of Interest Statement

The authors declare that the research was conducted in the absence of any commercial or financial relationships that could be construed as a potential conflict of interest.
